# Smoking remains associated with education after controlling for social background and genetic factors in a study of 18 twin cohorts

**DOI:** 10.1038/s41598-022-17536-x

**Published:** 2022-07-31

**Authors:** Karri Silventoinen, Maarit Piirtola, Aline Jelenkovic, Reijo Sund, Adam D. Tarnoki, David L. Tarnoki, Emanuela Medda, Lorenza Nisticò, Virgilia Toccaceli, Chika Honda, Fujio Inui, Rie Tomizawa, Mikio Watanabe, Norio Sakai, Margaret Gatz, David A. Butler, Jooyeon Lee, Soo Ji Lee, Joohon Sung, Carol E. Franz, William S. Kremen, Michael J. Lyons, Catherine A. Derom, Robert F. Vlietinck, Ruth J. F. Loos, Per Tynelius, Finn Rasmussen, Nicholas G. Martin, Sarah E. Medland, Grant W. Montgomery, Ingunn Brandt, Thomas S. Nilsen, Jennifer R. Harris, Jessica Tyler, John L. Hopper, Patrik K. E. Magnusson, Nancy L. Pedersen, Anna K. Dahl Aslan, Juan R. Ordoñana, Juan F. Sánchez-Romera, Lucia Colodro-Conde, Esther Rebato, Dongfeng Zhang, Zengchang Pang, Qihua Tan, Judy L. Silberg, Hermine H. Maes, Dorret I. Boomsma, Thorkild I. A. Sørensen, Tellervo Korhonen, Jaakko Kaprio

**Affiliations:** 1 Department of Social Research, Faculty of Social Sciences, University of Helsinki, P.O. Box 18, 00014 Helsinki, Finland; 2 Center for Twin Research, Osaka University Graduate School of Medicine, Osaka, Japan; 3 UKK Institute – Centre for Health Promotion Research, Tampere, Finland; 4 Institute for Molecular Medicine Finland FIMM, University of Helsinki, Helsinki, Finland; 5 Department of Physiology, Faculty of Medicine and Nursing, University of the Basque Country, Leioa, Spain; 6 Department of Public Health, University of Helsinki, Helsinki, Finland; 7 Institute of Clinical Medicine, University of Eastern Finland, Kuopio, Finland; 8 Medical Imaging Centre, Semmelweis University, Budapest, Hungary; 9 Hungarian Twin Registry, Budapest, Hungary; 10 Istituto Superiore di Sanità - Centre for Behavioural Sciences and Mental Health, Rome, Italy; 11 School of Nursing, Shiga University of Medical Science, Osaka, Japan; 12 Faculty of Health Science, Kio University, Nara, Japan; 13 Center for Economic and Social Research, University of Southern California, Los Angeles, CA USA; 14 Department of Medical Epidemiology and Biostatistics, Karolinska Institutet, Stockholm, Sweden; 15 The National Academies of Sciences, Engineering, and Medicine, Washington, DC USA; 16 Department of Epidemiology, School of Public Health, Seoul National University, Seoul, South Korea; 17 Institute of Health and Environment, Seoul National University, Seoul, South Korea; 18 Department of Psychiatry, University of California, San Diego, CA USA; 19 VA San Diego Center of Excellence for Stress and Mental Health, La Jolla, CA USA; 20 Department of Psychological and Brain Sciences, Boston University, Boston, MA USA; 21 Centre of Human Genetics, University Hospitals Leuven, Leuven, Belgium; 22 Department of Obstetrics and Gynaecology, Ghent University Hospitals, Ghent, Belgium; 23 The Charles Bronfman Institute for Personalized Medicine, The Mindich Child Health and Development Institute, Icahn School of Medicine at Mount Sinai, New York, NY USA; 24 Department of Global Public Health, Karolinska Institutet, Stockholm, Sweden; 25 Genetic Epidemiology Department, QIMR Berghofer Medical Research Institute, Brisbane, Australia; 26 Institute for Molecular Bioscience, The University of Queensland, Brisbane, Australia; 27 Division of Health Data and Digitalization, Norwegian Institute of Public Health, Oslo, Norway; 28 Center for Fertility and Health, The Norwegian Institute of Public Health, Oslo, Norway; 29 Centre for Epidemiology and Biostatistics, Twins Research Australia, The University of Melbourne, Melbourne, VIC Australia; 30 School of Health Sciences, University of Skövde, Skövde, Sweden; 31 Department of Human Anatomy and Psychobiology, University of Murcia, Murcia, Spain; 32 IMIB-Arrixaca, Murcia, Spain; 33 Department of Genetics, Physical Anthropology and Animal Physiology, University of the Basque Country UPV/EHU, Leioa, Spain; 34 Department of Public Health, Qingdao University Medical College, Qingdao, China; 35 Department of Noncommunicable Diseases Prevention, Qingdao Centers for Disease Control and Prevention, Qingdao, China; 36 Epidemiology, Biostatistics and Biodemography, Department of Public Health, University of Southern Denmark, Odense, Denmark; 37 Department of Human and Molecular Genetics, Virginia Institute for Psychiatric and Behavioral Genetics, Virginia Commonwealth University, Richmond, VA USA; 38 Department of Human and Molecular Genetics, Psychiatry and Massey Cancer Center, Virginia Commonwealth University, Richmond, VA USA; 39 Netherlands Twin Register, Department of Biological Psychology, Vrije Universiteit, Amsterdam, Amsterdam, The Netherlands; 40 Faculty of Health and Medical Sciences, Novo Nordisk Foundation Centre for Basic Metabolic Research, University of Copenhagen, Copenhagen, Denmark; 41 Department of Public Health (Section of Epidemiology), Faculty of Health and Medical Sciences, University of Copenhagen, Copenhagen, Denmark

**Keywords:** Genetics, Risk factors

## Abstract

We tested the causality between education and smoking using the natural experiment of discordant twin pairs allowing to optimally control for background genetic and childhood social factors. Data from 18 cohorts including 10,527 monozygotic (MZ) and same-sex dizygotic (DZ) twin pairs discordant for education and smoking were analyzed by linear fixed effects regression models. Within twin pairs, education levels were lower among the currently smoking than among the never smoking co-twins and this education difference was larger within DZ than MZ pairs. Similarly, education levels were higher among former smoking than among currently smoking co-twins, and this difference was larger within DZ pairs. Our results support the hypothesis of a causal effect of education on both current smoking status and smoking cessation. However, the even greater intra-pair differences within DZ pairs, who share only 50% of their segregating genes, provide evidence that shared genetic factors also contribute to these associations.

## Introduction

Smoking is globally the leading behavioral risk factor for health^1^. Even though the prevalence of smoking has decreased over the last decades, in 2015, it still caused 6.4 million annual deaths globally^2^. Current smoking patterns also show clear and persistent socio-economic inequalities such that individuals in lower social positions tend more often to be smokers and more rarely quit smoking than those in higher social positions^3^. Thus, smoking has important effects on socioeconomic inequalities in mortality; this effect is larger in men but has increased more in women during the last decades^4^. Prevention of smoking initiation and promotion of smoking cessation would thus be important elements of policy interventions to improve population health in general, and also to decrease socio-economic health inequalities.

Even when socio-economic inequalities in smoking are well known^3^, their causes are still under debate. The most straightforward explanation is that higher education leads to lower probability to start smoking, or higher probability of smoking cessation in smokers, because of, for example, better health literacy^5^. However, other factors may also explain this association. First, childhood social and family factors may affect both smoking initiation and education. Since parental smoking increases the risk of smoking initiation among offspring^6^ and parental education is associated with offspring education^7^, the correlation between education and smoking may be transmitted through generations. There is clear evidence that maternal smoking during pregnancy is associated with lower offspring IQ^8^ and poorer school performance^9^. However, these associations may not reflect a causal link from prenatal nicotine exposure to cognitive ability but rather unmeasured maternal characteristics. The association between maternal smoking and offspring IQ faded when adjusted for maternal IQ^8^. Furthermore, children not exposed to maternal smoking during pregnancy had poorer school performance if the mother had smoked during another pregnancy^9^. Smoking is also generally initiated in adolescence^10^ when the influence of peers strongly increases^11^. If neighborhoods are stratified by socio-economic status, the educational level of peers can have an impact on both education and smoking initiation^12^.

Second, it is possible that genetic effects contribute to this association. Previous twin studies have shown moderate heritability for both smoking^13^ and education^14^. Genome-wide-association (GWA) studies have identified a large number of single-nucleotide-polymorphisms (SNP) associated with smoking behavior traits^15,16^ and education^17^, many of them expressed in brain tissue. It is possible that some of the genetic variation is shared between smoking and education because of pleiotropic effects or common risk factors. For example, attention-deficit/hyperactivity disorder (ADHD) is associated with higher probability of smoking^18^, and untreated ADHD is also associated with worse academic achievements^19^. Since there is genetic liability to ADHD risk^20^, it can contribute to a genetic correlation between education and smoking. There is some direct evidence for this from a study that found four SNPs common to smoking and education^16^. The genetic correlation based on all SNPs in the UK Biobank was found to be − 0.56 between education and current smoking^21^. However, the genetic correlations can also arise because of genetic nurture effects, since non-transmitted parental alleles of an educational polygenic risk score were also found to be associated with smoking in the offspring^22^.

In the present study, we aimed to analyze the association between smoking status and educational attainment in a large multinational cohort of twins. The twin study design offers a unique natural experiment to control for childhood social background and genetic factors. While monozygotic (MZ) twins are virtually genetically identical, dizygotic (DZ) twins share, on average, half of their segregating genes. However, both MZ and DZ twins share intrauterine environmental exposures, such as nicotine exposure because of maternal smoking, and childhood environmental factors, such as childhood home, neighborhood and common schools and friends. On the other hand, they may have different friends and teachers or have other unique environmental exposures. Therefore, we can formulate three conditions: (1) if the association between education and smoking is found only in individual level analyses, but not within smoking-discordant MZ and DZ pairs, it indicates that the association is not causal, but likely explained by childhood family background or genetic factors; (2) if similar associations are found in individual level analyses and within pair analyses of MZ and DZ twins, it may indicate a causal association between education and smoking or the influence of environmental factors unique to each co-twin; and (3) if the association is found only in individual level and within DZ but not within MZ pairs, it suggests that genetic factors account for this association^23^. Our large database allows us to analyze how these associations have changed during the last five decades when the global prevalence of smoking has decreased.

## Results

Table 1 presents the descriptive statistics of the study cohort. The earliest measurement period (1960–1969) was exceptional since it included only men and the educational level was higher than in the later periods. This is because this period included only one cohort (NAS-NRC Twin Cohort) having US veterans of the Second World War. In the later periods including both men and women, men were more commonly smokers than women. This sex difference decreased somewhat when coming in the latest period (2000–2012) due to the decreasing prevalence of smoking among men. The proportion of never smokers increased over time among men, but no clear trend was observed for women. In men, never smokers had systematically higher education than current and former smokers, but in women, these differences were less consistent.

**Table 1:** Number of observations and descriptive statistics of educational years by smoking status, measurement year and sex.

	Number of observations		Never smokers^a^			Current smokers^a^			Former smokers^a^		
	All individuals	Discordant pairs	%	Educational years		%	Educational years		%	Educational years	
				Mean	SD		Mean	SD		Mean	SD
**Men**											
1960–1969	5086	754	31	14.5	3.05	41	13.1	2.95	28	13.7	3.05
1970–1979	8246	867	32	8.7	4.10	40	7.7	3.10	28	8.3	3.62
1980–1989	17,204	1848	36	12.1	4.55	31	10.0	3.88	33	11.5	4.31
1990–1999	8378	922	44	11.4	4.34	26	10.3	3.99	35	10.4	4.37
2000–2012	13,764	1435	48	13.3	3.81	24	11.9	3.71	29	12.2	4.03
**Women**											
1970–1979	8366	537	69	7.7	3.11	20	8.1	2.95	11	8.7	3.52
1980–1989	19,332	1526	56	10.9	4.16	26	10.9	3.55	18	11.5	3.86
1990–1999	11,138	1114	58	10.8	4.14	22	10.6	3.88	20	11.3	4.03
2000–2012	17,614	1524	60	13.1	4.13	20	12.2	3.87	20	12.7	3.97

Twin cohorts from the following countries were included in the analyses: Australia (Queensland Twin Register, Twin Research Australia), China (Qingdao Twin Registry), Belgium (East Flanders Prospective Twin Survey), Finland (FinnTwin12, FinnTwin16, Finnish Older Cohort), Hungary (Hungarian Twin Registry), Italy (Italian Twin Registry), Japan (Osaka University Aged Twin Registry), Norway (Norwegian Twin Registry), South Korea (Korean Twin-Family Register), Spain (Murcia Twin Registry), Sweden (Swedish Twin Registry, Swedish Young Male Twins Study) and USA (Mid Atlantic Twin Registry, NAS-NRC Twin Cohort, Vietnam Era Twin Registry).

^a^Calculated within all individuals.

Table 2 presents the regression coefficients of educational years of current and former smokers as compared to never smokers by measurement period and sex including all twins shown in Table 1. Smokers and former smokers had lower education than never smokers in men and women; the only exception was the earliest period for which we had information on women (1970–1979) where female former smokers were slightly more educated than never smokers. Educational differences were larger in men than in women, but these sex differences changed over the measurement periods. In the period of 1970–1979, the difference of educational years between current smokers and never smokers was 0.71 years (95% CI 0.45, 0.96) larger in men as compared to women, but this sex difference decreased to 0.20 years (95% CI − 0.01, 0.40) in the latest period of 2000–2012. When comparing former smokers to never smokers, the decline in the rate of educational differences between men and women over the measurement periods was even larger: 0.92 (95% CI 0.59, 1.25) educational years in 1970–1979 and 0.22 (95% CI 0.01, 0.42) educational years in 2000–2012.

**Table 2:** Educational years of current smokers and former smokers as compared to never smokers by sex and measurement year.

Measurement years	Men			Women			*p*-value of sex interactions
	β	95% confidence intervals		β	95% confidence intervals		
		LL	UL		LL	UL	
**Current smokers**							
1960–1969	− 1.41	− 1.64	− 1.19	NA			NA
1970–1979	− 1.03	− 1.22	− 0.84	− 0.30	− 0.48	− 0.13	< 0.0001
1980–1989	− 1.46	− 1.59	− 1.33	− 0.83	− 0.95	− 0.72	< 0.0001
1990–1999	− 0.98	− 1.18	− 0.79	− 0.91	− 1.08	− 0.75	0.172
2000–2012	− 1.34	− 1.49	− 1.19	− 1.09	− 1.23	− 0.95	0.031
**Former smokers**							
1960–1969	− 0.76	− 1.00	− 0.52	NA			NA
1970–1979	− 0.43	− 0.63	− 0.23	0.51	0.26	0.77	< 0.0001
1980–1989	− 0.74	− 0.87	− 0.61	− 0.18	− 0.31	− 0.06	< 0.0001
1990–1999	− 0.36	− 0.58	− 0.15	− 0.01	− 0.19	0.16	0.009
2000–2012	− 0.63	− 0.78	− 0.47	− 0.42	− 0.55	− 0.28	0.036

Adjusted for age, birth cohort and twin cohort.

After the individual-based association analyses, we analyzed how education differed within twin pairs discordant for smoking status. We started these analyses by identifying pairs in which one twin was a never smoker and his/her co-twin was a current smoker (Table 3). Within both MZ and same-sex DZ twin pairs, the never smoking twin had a higher level of education than the currently smoking co-twin. No consistent differences were found between men and women or between the measurement periods. However, the within pair educational difference was generally larger within discordant DZ twins than within discordant MZ twins. In men, this zygosity difference was found in all measurement periods: in the pooled data it was − 0.19 (95% CI − 0.28, − 0.09) educational years within MZ pairs and − 0.55 (95% CI − 0.64, − 0.46) educational years within DZ twin pairs. In women, the results based on analyses stratified by the measurement period were less systematic. However, also in women, the difference in educational years was smaller within MZ twin pairs (− 0.17 95% CI − 0.26, − 0.08) as compare to DZ twin pairs (− 0.38 95% CI − 0.44, − 0.26) in the pooled data. The zygosity difference was statistically significant in men (*p* < 0.0001) and women (*p* = 0.008) in the pooled data, indicating that differences within DZ discordant pairs were larger than within MZ discordant pairs.

**Table 3:** Educational years in current smokers as compared to never smokers within discordant twin pairs by measurement year, zygosity and sex.

Measurement years	MZ twins			Same-sex DZ twins			*p*-value of zygosity interaction
	β	95% confidence intervals		β	95% confidence intervals		
		LL	UL		LL	UL	
**Men**							
1960–1969	− 0.22	− 0.52	0.07	− 0.89	− 1.23	− 0.54	0.005
1970–1979	− 0.16	− 0.44	0.12	− 0.39	− 0.61	− 0.16	0.295
1980–1989	− 0.19	− 0.38	0.00	− 0.76	− 0.94	− 0.58	< 0.0001
1990–1999	− 0.10	− 0.43	0.24	− 0.41	− 0.73	− 0.09	0.221
2000–2012	− 0.26	− 0.47	− 0.05	− 0.60	− 0.84	− 0.35	0.045
**Women**							
1970–1979	− 0.25	− 0.48	− 0.01	0.03	− 0.17	0.24	0.136
1980–1989	− 0.18	− 0.32	− 0.03	− 0.44	− 0.60	− 0.28	0.029
1990–1999	− 0.42	− 0.69	− 0.14	− 0.43	− 0.69	− 0.16	0.964
2000–2012	− 0.11	− 0.32	0.09	− 0.57	− 0.79	− 0.34	0.004

We then conducted the corresponding analyses for twin pairs with a former smoker and a never smoker co-twin (Table 4). The formerly smoking twin had generally lower education than the never smoking co-twin, but in most of the periods, the difference was not statistically significant. However, in the pooled analyses of men, the difference in education was − 0.12 (95% CI − 0.21, − 0.03) years within MZ twin pairs and − 0.22 (95% CI − 0.31, − 0.13) years within DZ twin pairs. In women, we found a small and statistically non-significant difference within MZ twins (− 0.06 95% CI − 0.14, 0.03) and somewhat larger difference within DZ twins (− 0.10 95% CI − 0.18, − 0.01). The zygosity differences were thus in the same direction as when comparing never and current smoking co-twins (Table 3), but they were not statistically significant in men (*p* = 0.12) or in women (*p* = 0.50), even in these pooled analyses.

**Table 4:** Educational years in former smokers as compared to never smokers within discordant twin pairs by measurement year, zygosity and sex.

Measurement years	MZ twins			Same-sex DZ twins			*p*-value of zygosity interaction
	β	95% confidence intervals		β	95% confidence intervals		
		LL	UL		LL	UL	
**Men**							
1960–1969	− 0.16	− 0.45	0.14	− 0.49	− 0.85	− 0.13	0.166
1970–1979	− 0.08	− 0.36	0.19	− 0.01	− 0.24	0.22	0.726
1980–1989	− 0.03	− 0.20	0.13	− 0.38	− 0.55	− 0.20	0.012
1990–1999	− 0.09	− 0.40	0.22	− 0.02	− 0.32	0.28	0.778
2000–2012	− 0.21	− 0.41	− 0.02	− 0.20	− 0.43	0.03	0.936
**Women**							
1970–1979	− 0.28	− 0.55	− 0.02	0.08	− 0.17	0.32	0.091
1980–1989	− 0.15	− 0.30	− 0.01	− 0.14	− 0.31	0.03	0.896
1990–1999	− 0.11	− 0.37	0.15	0.00	− 0.24	0.25	0.560
2000–2012	0.08	− 0.10	0.26	− 0.20	− 0.41	0.01	0.053

The last set of discordant pair analyses was conducted based on pairs with a former smoker and a current smoking co-twin (Table 5). The formerly smoking twin had generally higher education than the currently smoking co-twin, and the associations were stronger within DZ than within MZ twin pairs. This was confirmed in the pooled analyses where the association was weaker within MZ pairs (0.07 95% CI − 0.01, 0.14 difference in educational years in men and 0.12 95% CI 0.03, 0.20 educational years in women) as compared to DZ pairs (0.32 95% CI 0.24, 0.40 and 0.28 95% CI 0.15, 0.34 difference educational years, respectively); the zygosity difference was statistically significant in men (*p* < 0.0001) and in women (*p* = 0.04).

**Table 5:** Educational years in former smokers as compared to current smokers within discordant twin pairs by measurement year, zygosity and sex.

Measurement years	MZ twins			Same-sex DZ twins			*p*-value of zygosity interaction
	β	95% confidence intervals		β	95% confidence intervals		
		LL	UL		LL	UL	
**Men**							
1960–1969	0.07	− 0.17	0.30	0.39	0.09	0.70	0.095
1970–1979	0.07	− 0.15	0.30	0.38	0.17	0.58	0.088
1980–1989	0.16	0.01	0.31	0.39	0.22	0.55	0.063
1990–1999	0.01	− 0.29	0.31	0.39	0.09	0.68	0.104
2000–2012	0.04	− 0.15	0.24	0.40	0.16	0.63	0.028
**Women**							
1970–1979	− 0.04	− 0.31	0.23	0.04	− 0.21	0.30	0.712
1980–1989	0.02	− 0.11	0.15	0.30	0.13	0.47	0.015
1990–1999	0.31	0.05	0.57	0.43	0.16	0.71	0.542
2000–2012	0.19	− 0.01	0.39	0.37	0.14	0.60	0.261

Finally, we conducted a sensitivity analysis for individual level associations using only twins from discordant pairs (Supplementary Table 1). The point estimates were close to the analyses using all data (Table 1), but the CIs were wider because of the smaller sample size. Noteworthy, the point estimates were systematically larger than in the within-pair analyses both for MZ and DZ twins (Tables 3, 4, 5).

## Discussion

In this large study of pooled twin cohorts with information on smoking status and education, we found that the changes in smoking prevalence closely followed the epidemic pattern presented by Lopez and co-authors already in 1994^24^. Smoking prevalence started to decrease in men during the 1960s–1980s when it still increased in women. During the 1990s and 2000s, the smoking prevalence decreased both in men and women, but the rate was more rapid in men. Sex differences were also found when studying the association between smoking and education. Educational differences between never smokers as compared to both current smokers and former smokers were larger in men than in women, but this sex difference decreased from the 1970s to the 2000s. This is congruent with the historical development when smoking in women became first more common in upper social classes and only at the later phase of the epidemic became more prevalent in lower social classes^25^. An advantage of our study is that we were able to analyze this pattern over 50 years, which has rarely been done on a global scale.

The main research aim in our study was to investigate the causality of the association between educational attainment and smoking. Our results are consistent with the hypothesis of a causal association between level of education with current smoking and with smoking cessation. For current smoking, we observed that the currently smoking co-twin had lower education than the never smoking co-twin in twin pairs discordant for smoking. Correspondingly, for smoking cessation, the formerly smoking co-twin was better educated than the currently smoking co-twin within discordant MZ twin pairs. This may indicate that the higher education leads to non-smoking because of, for example, better health literacy^5^. However, especially when considering smoking initiation, also the reverse causal direction is possible since smoking can lead to attention problems^26^. These associations can also be bidirectional as suggested by a Finnish longitudinal study of educational achievement and smoking from 12 years of age to early adulthood^27^. Our cross-sectional study design focusing only on adults cannot answer to the question of the direction of causality. This emphasizes the need of longitudinal studies with detailed measures of smoking and school performance from childhood to adulthood.

Since MZ co-twins are virtually genetically identical and also share prenatal risk factors such as nicotine exposure during pregnancy, common family background and many other childhood environmental factors, they create an almost perfect natural experimental design to study the causality hypothesis. The advantage of twin studies is that they allow to control for environmental factors shared by co-twins in addition to genetic factors. Previous twin studies have shown that shared environmental factors can be important for smoking initiation but are less so for smoking persistence and smoking amount and there is hardly any evidence that shared environment affects nicotine dependence^28^. The effect of shared environment on smoking initiation can reflect the role of childhood family in adolescence when smoking is typically started^10^. Further, there is strong evidence that shared environmental factors are important for educational achievement^14^. Thus, when considering the association between smoking and education, it is important to consider also the role of family background and other early environmental factors in addition to genetic factors. This was emphasized by a recent GWA study finding that the genetic correlation between education and smoking decreased if considering only genetic differences within siblings suggesting that a part of the genetic correlation at population level is mediated by family environment^29^.

Even when socio-economic differences in smoking are well established^3^, studies capable of assessing causal inference are still rare. A Mendelian randomization study found that genetic variants related to education predicted smoking behavior^30^; however, the Mendelian randomization could not exclude the possibility that this association is caused by pleiotropy, a genetic nurture effect or a third factor affecting both smoking and education. A Swedish study used month-of-birth as an instrumental variable of academic achievement since older students in the class have, on average, better academic performance than younger students^31^. According to the hypothesis, a birth month in autumn was associated with a higher risk of smoking. In this study, analyses of discordant co-relatives (cousins, siblings and MZ twins) also provided suggestive evidence for a causal association; however, the number of MZ twins was small and the associations within such pairs were not statistically significant. A US study of discordant sibling pairs found associations between educational attainment and several lifetime smoking behavior traits (number of cigarettes smoked, number of 24-h quit attempts, number of 3-month abstinence periods and smoking cessation), but the results were not statistically significant^32^. This demonstrates the need of large cohorts to identify enough discordant pairs to get reliable results.

Our study provides evidence of the role of shared genetic factors underlying the associations of educational attainment with current smoking and smoking cessation. We found that these associations were systematically strongest among individual-based analyses, followed by those within discordant DZ pairs, and were weakest within discordant MZ pairs. This suggests that the associations of education with smoking and cessation are partly accounted for genetic factors. There is previous evidence based on GWA studies on the overlap of SNPs associated with education and smoking^16,21^. However, this overlap can also reflect nature-nurture effects as well as mediation mechanisms^22^. Thus, these previous results do not directly show that there are common genetic variants shared by both education and smoking. Our results provide additional evidence for this hypothesis since the nature-nurture effect cannot explain the stronger associations within DZ than MZ pairs. The nature of this genetic overlap is not clear. Since many of the SNPs associated with smoking^15,16^ and education^17^ are expressed in the brain tissue, this overlap can arise from pleiotropic effects. However, it can also reflect the influence of common background factors, such as personality and psychopathology, affected by genetic factors. Mediation may also be a mechanism, i.e. genes that predispose to smoking act through smoking’s direct effects on brain function and appear as genes associated with education^33^. To resolve these alternative explanations, the association between education and a polygenic risk score for smoking, as a measure of genetic susceptibility, could be analyzed among never smokers. This design would eliminate the possibility of mediation.

Our study has important strengths but also limitations. The main strength is that we have a large twin dataset covering five decades of data collection. Thus, we can analyze how the associations between education and smoking have changed over the smoking epidemic using the unique natural experiment of twins controlling for genetic factors and childhood environment. Our main limitation is that we have only information on smoking status but not on age when smoking was initiated, number of cigarettes smoked, nicotine dependence or other smoking behaviors, such as use of other nicotine products. This information provided more information on the background of the observed associations. Further, our dataset is biased toward affluent societies following a Westernized life-style and only three countries with mainly non-Caucasian populations are represented, all of them from East Asia. Thus, it would be very important to promote data collection in now underrepresented areas of the world. Finally, it has to be noted that even though the discordant twin pair design is a powerful tool to control unobserved genetic and environmental confounders, it also has limitations^34^. Most notable, the association within discordant twin pairs includes unshared environmental influences and correlated measurement errors in addition to causal effects. For example, there can be friends or environmental exposures affecting only one twin. If these environmental factors affect both smoking and education, their effect is seen as the association between smoking and education within co-twins. It is also possible that some individuals report both higher education and non-smoking if they want to give, for example, better impression of themselves. Thus, even when our results are consistent with the hypothesis of the causal association between education and smoking, it would be important to test this hypothesis using other types of study design, such as detailed longitudinal studies of adolescents and young adults.

In conclusion, the results of this large study of pooled twin cohorts are consistent with the hypothesis of a causal association between education and smoking. However, we also found evidence that common genetic factors can explain part of the association between education and smoking. More detailed information on factors mediating these associations would be important to find ways to decrease socio-economic inequalities in smoking.

## Data and methods

The data were derived from the international CODATwins (COllaborative project of Development of Anthropometrical measures in Twins) database described in detail elsewhere^35,36^. The CODATwins project was established to pool together all twin data in the world having information of height and weight. All participants were volunteers and they or their parents gave informed consent when participating in their original studies. Only a limited set of observational variables and anonymized data were delivered to the data management center at University of Helsinki. The pooled analysis was approved by the ethical committee of Department of Public Health, University of Helsinki, and the methods were carried out in accordance with the approved guidelines. In this study, we used cohorts which had provided additional information on education and smoking status and had at least 50 twin individuals eligible to the study. Different educational classifications used in collaborating twin cohorts were transformed into educational years as described in detail elsewhere^37^. The educational years varied from 0 (illiteracy/no formal education) to 22 (doctoral level education). Smoking status was classified as never smokers, current smokers and former smokers.

Together, 18 twin cohorts were included in this study (the names of participating cohorts are given in the footnote of Table 1). We excluded those younger than 25 years of age since they may not yet have finalized their education, and also those 70 years of age or older since the elderly population is increasingly selected for their smoking status because of higher mortality of smokers over the years. Further, since the main emphasis is on within pair analyses, we excluded opposite-sex twin pairs because of the large differences between men and women in smoking patterns and education in earlier birth cohorts. In the final study cohort, we had 102,537 twin individuals of whom 42% were MZ twins and 50% women. Within these twins, we had 10,527 twin pairs (35% MZ, 45% women) where co-twins had different smoking status and education thus informative when studying within pair variation. We conducted analyses in 10-year periods based on the measurement year to analyze how the association between smoking and education had changed during the five decades from the 1960s to the 2000s. Some of the twin cohorts are longitudinal having repeated measures for some individuals. However, we included only one measure from each twin per measurement period and stratified all analyses by it to confirm the statistical independence of observations for all statistical models. Further, we selected only paired observations in each period to confirm that individual and within pair analyzes are based on the same data. Together, we had 109,128 observations over the measurement years. The mean age was somewhat higher in the latest cohorts but no differences in mean ages were found between men and women (Supplementary Table 2).

When studying individual level associations, we used the linear regression model for each age-, sex, and zygosity specific group after the effect of intra-pair correlations (i.e., sampling twin pairs rather than independent individuals) on standard errors and confidence intervals (CI) was taken into account. In order to analyze the association between smoking and education within co-twins, we fitted separate linear fixed-effect regression models and took into account the co-twins with a set of dummy-variables^34^. In practice, the model compares the co-twins to each other and removes the confounding influences of all fixed genetic and social characteristics that the co-twins share. Comparing the estimates from within pair analyses to individual level analyses can produce information on the role of shared genetic and environmental factors on the association between smoking and education. All analyses were conducted by Stata/MP 16.0 for Windows statistical software (StataCorp, College Station, TX, USA).

## Supplementary Information

Supplementary Tables.
