# Supplementary material for: Smoking remains associated with education after controlling for social background and genetic factors in a study of 18 twin cohorts
Source: Sci Rep. 2022 Jul 31;12:13148. doi: 10.1038/s41598-022-17536-x (PMC9339539; doi:10.1038/s41598-022-17536-x)
Supplement: Supplementary file 1 — Supplementary Tables. [file 41598_2022_17536_MOESM1_ESM.docx]

Supplementary table 1. Proportions of participants and educational years of current smokers and former smokers as compared to never smokers at individual level by sex and measurement year in twins from pairs discordant for education and smoking.^1^

| Measurement years | Men | | | | Women | | | | p-value of sex interactions |
| --- | --- | --- | --- | --- | --- | --- | --- | --- | --- |
|  | % | β | 95% confidence intervals | | % | β | 95% confidence intervals | |  |
|  |  |  | LL | UL |  |  | LL | UL |  |
| Current smokers |  |  |  |  |  |  |  |  |  |
| 1960–1969 | 42 | -1.30 | -1.70 | -0.90 | NA | NA |  |  | NA |
| 1970–1979 | 38 | -0.77 | -1.22 | -0.32 | 38 | -0.12 | -0.55 | 0.30 | 0.059 |
| 1980–1989 | 31 | -1.35 | -1.63 | -1.07 | 33 | -0.62 | -0.88 | -0.36 | <0.001 |
| 1990–1999 | 29 | -0.53 | -0.96 | -0.10 | 30 | -0.77 | -1.13 | -0.41 | 0.499 |
| 2000–2012 | 30 | -1.25 | -1.55 | -0.95 | 28 | -0.76 | -1.05 | -0.48 | 0.011 |
|  |  |  |  |  |  |  |  |  |  |
| Former smokers |  |  |  |  |  |  |  |  |  |
| 1960–1969 | 34 | -0.89 | -1.30 | -0.49 | NA | NA |  |  | NA |
| 1970–1979 | 33 | -0.34 | -0.82 | 0.14 | 23 | 0.29 | -0.27 | 0.84 | 0.010 |
| 1980–1989 | 38 | -0.60 | -0.88 | -0.33 | 32 | -0.16 | -0.43 | 0.10 | 0.007 |
| 1990–1999 | 37 | -0.27 | -0.72 | 0.17 | 34 | 0.07 | -0.27 | 0.41 | 0.239 |
| 2000–2012 | 37 | -0.55 | -0.83 | -0.27 | 35 | -0.08 | -0.34 | 0.19 | 0.009 |

^1^Adjusted for age, birth cohort and twin cohort

Supplementary table 2. Mean, standard deviation and range of age by birth cohort and sex.

| Measurement years | Men | | | | Women | | | |
| --- | --- | --- | --- | --- | --- | --- | --- | --- |
|  | Mean | SD | Lower age | Upper age | Mean | SD | Lower age | Upper age |
| 1960–1969 | 44 | 2.89 | 40 | 50 | NA | NA | NA | NA |
| 1970–1979 | 40 | 11.21 | 25 | 69 | 41 | 12.0 | 25 | 69 |
| 1980–1989 | 44 | 14.01 | 25 | 69 | 41 | 13.0 | 25 | 69 |
| 1990–1999 | 47 | 12.89 | 25 | 69 | 48 | 12.5 | 25 | 69 |
| 2000–2012 | 47 | 12.17 | 25 | 69 | 46 | 12.1 | 25 | 69 |
